# Diagnostic and prognostic biomarkers for progressive fibrosing interstitial lung disease

**DOI:** 10.1371/journal.pone.0283288

**Published:** 2023-03-17

**Authors:** Mayuko Watase, Takao Mochimaru, Honomi Kawase, Hiroyuki Shinohara, Shinobu Sagawa, Toshiki Ikeda, Shota Yagi, Hiroyuki Yamamura, Emiko Matsuyama, Masanori Kaji, Momoko Kurihara, Midori Sato, Kohei Horiuchi, Risa Watanabe, Shigenari Nukaga, Kaoru Irisa, Ryosuke Satomi, Yoshitaka Oyamada

**Affiliations:** 1 Department of Respiratory Medicine, National Hospital Organization Tokyo Medical Center, Tokyo, Japan; 2 Department of Allergy, National Hospital Organization Tokyo Medical Center, Tokyo, Japan; Rutgers Biomedical and Health Sciences, UNITED STATES

## Abstract

No biomarkers have been identified in bronchoalveolar lavage fluid (BALF) for predicting fibrosis progression or prognosis in progressive fibrosing interstitial lung disease (PF-ILD). We investigated BALF biomarkers for PF-ILD diagnosis and prognosis assessment. Overall, 120 patients with interstitial pneumonia who could be diagnosed with PF-ILD or non PF-ILD were enrolled in this retrospective study. PF-ILD was diagnosed according to Cottin’s definition. All patients underwent bronchoscopy and BALF collection. We evaluated blood and BALF parameters, high-resolution computed tomography (HRCT) patterns, and spirometry data to identify factors influencing PF-ILD diagnosis and prognosis. On univariate logistic analysis, age, sex, the BALF white blood cell fraction (neutrophil, lymphocyte, eosinophil, and neutrophil-to-lymphocyte ratio), BALF flow cytometric analysis (CD8), and an idiopathic pulmonary fibrosis/usual interstitial pneumonia pattern on HRCT were correlated with PF-ILD diagnosis. Multivariate logistic regression analysis revealed that sex (male), age (cut-off 62 years, area under the curve [AUC] 0.67; sensitivity 0.80; specificity 0.47), white blood cell fraction in BALF (NLR, neutrophil, and lymphocyte), and CD8 in BALF (cut-off 34.2; AUC 0.66; sensitivity, 0.74; specificity, 0.62) were independent diagnostic predictors for PF-ILD. In BALF, the NLR (cut-off 8.70, AUC 0.62; sensitivity 0.62; specificity 0.70), neutrophil count (cut-off 3.0, AUC 0.59; sensitivity 0.57; specificity 0.63), and lymphocyte count (cut-off 42.0, AUC 0.63; sensitivity 0.77; specificity 0.53) were independent diagnostic predictors. In PF-ILD patients (n = 77), lactate dehydrogenase (cut-off 275, AUC 0.69; sensitivity 0.57; specificity 0.78), Krebs von den Lungen-6 (cut-off 1,140, AUC 0.74; sensitivity 0.71; specificity 0.76), baseline forced vital capacity (FVC) (cut-off 1.75 L, AUC 0.71; sensitivity, 0.93; specificity, 0.46), and BALF neutrophil ratio (cut-off 6.0, AUC 0.72; sensitivity 0.79; specificity 0.80) correlated with death within 3 years. The BALF cellular ratio, particularly the neutrophil ratio, correlated with the diagnosis and prognosis of PF-ILD. These findings may be useful in the management of patients with interstitial pneumonia.

## Introduction

Interstitial lung disease (ILD) comprises heterogeneous subtypes of diffuse lung disorders [1, 2]. Idiopathic pulmonary fibrosis (IPF) is a typical progressive and fatal fibrotic ILD [3]. The prognosis of most non-IPF ILD cases is better than that of IPF cases; however, some non-IPF patients show rapid progression similar to those with IPF. Non-IPF ILDs with progressive fibrosing phenotypes include non-specific interstitial pneumonia, hypersensitivity pneumonia (HP), autoimmune ILDs, and sarcoidosis [4]. Recently, a subset of fibrosing ILD with a progressive course, despite conventional treatment, has been named progressive fibrosing ILD (PF-ILD) [5]. PF-ILD is characterized by worsening respiratory symptoms, a decline in lung function, and the extent of fibrosis on high-resolution computed tomography (HRCT). PF-ILD has a well-defined clinical phenotype, regardless of its cause. Mortality rates and lung function decline were found to be similar between patients with PF-ILD and those with IPF [1]. Because of early mortality, patients with PF-ILD require early diagnosis and prognostic markers to allow for implementation of precision medicine.

Although bronchoscopy is not necessary for patients with a usual interstitial pneumonia (UIP) pattern on CT scan, bronchoscopy remains necessary for patients with ILD in clinical settings [6]. Bronchoscopy is often required to rule out other conditions, such as respiratory infection. Moreover, factors in bronchoalveolar lavage fluid (BALF) have been used as markers of lower respiratory tract inflammation in many respiratory diseases.

Here, we sought to determine whether BALF contains biomarkers that may be useful for the diagnosis of PF-ILD and for predicting prognosis in these patients.

## Materials and methods

### Study population

**Fig 1:**
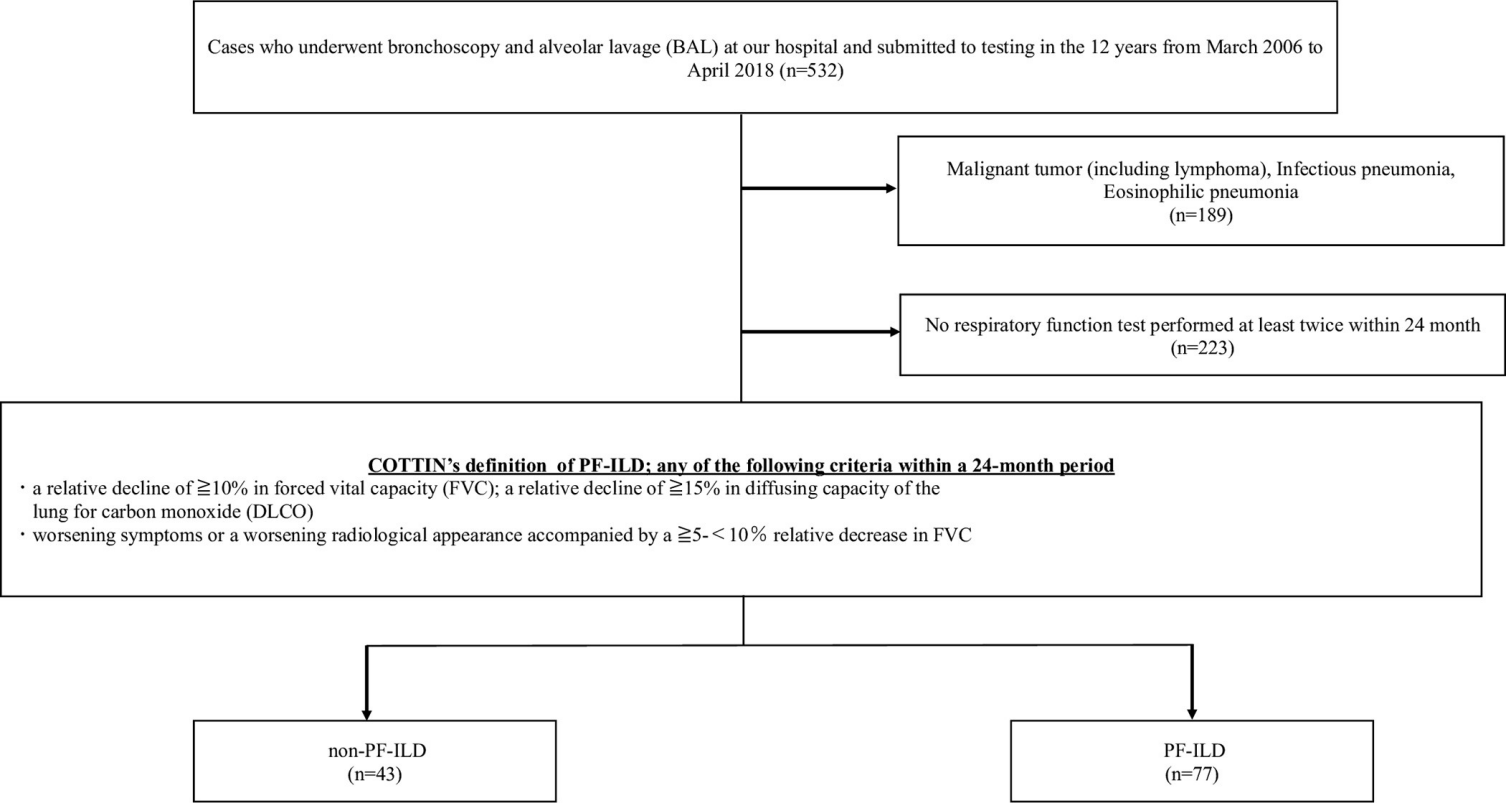
Population flow chart of the study cohort. Among 532 patients who underwent bronchoscopy and BAL, 189 patients with diseases not relevant to the objective of this study and 223 who did not undergo a respiratory function test were excluded. Eventually, 120 patients were included in the analysis. PF-ILD: progressive fibrosing interstitial lung disease; BAL: bronchoalveolar lavage.

Between March 2006 and April 2018, 532 patients underwent bronchoscopy and bronchoalveolar lavage (BAL) for disease evaluation at the Tokyo Medical Center, Tokyo, Japan (Fig 1). We collected the data of these 532 patients. Patients with malignant diseases, infectious pneumonia, or eosinophilic pneumonia were excluded. For this study, 120 patients with interstitial pneumonia who could be diagnosed with PF-ILD or non PF-ILD were enrolled in this retrospective study. PF-ILD was diagnosed according to Cottin’s definition [7], which was lung fibrosis along with one of the following criteria within 24 months of diagnosis despite receiving standard-of-care treatment: (1) relative decline in forced vital capacity (FVC) of ≥10% or relative decline of ≥15% in the diffusing capacity of the lungs for carbon monoxide (DLCO) or (2) worsening symptoms or radiological appearance, accompanied by a ≥5% but <10% relative decrease in FVC. Seventy-seven patients who met the above definition criteria were assigned to the PF-ILD group and the 43 who did not meet the definition criteria were assigned to the non PF-ILD group.

Informed consent was obtained in the form of opt-out. The Ethics Committee of the Tokyo Medical Center approved the study protocol. All aspects of the study conformed to the principles of the Declaration of Helsinki.

### BALF analysis

The patients underwent BAL using the method reported by Inomata et al. [8]. The affected segmental bronchus was identified on chest CT scan and was lavaged two or three times using 50-ml aliquots (total volume, 100 or 150 mL) of sterile 0.9% saline at room temperature through a wedged flexible fiberoptic bronchoscope. The BALF obtained was centrifuged at 1500 rpm for 5 min at 4°C to separate the supernatant from the cells. Cell pellets were counted in a hemocytometer, and Diff-Quik™ (International Reagents, Kobe, Japan)-stained smears were used to identify the differential profiles after cytospin preparation. Differential counts were performed by examining 300 cells using a standard light microscope.

The T lymphocyte subpopulations were determined using flow cytometry. The BALF samples were incubated with fluorescent monoclonal antibodies CD4 and CD8 (Beckman Coulter, Tokyo, Japan) and subsequently stained at room temperature in the dark for 15 min.

### Assessment of clinical parameters

Clinical and survival data of all patients were retrospectively collected from their medical records. Blood samples were collected within 1 month before bronchoscopy: peripheral blood fractions, biochemical tests including LDH and CRP, and markers of interstitial lung disease such as KL-6. Data on BMI and smoking status were collected during the physician’s interview within 1 month before bronchoscopy.

Since the purpose of this study was to search for predictors of PF-ILD from blood tests, BAL tests, and respiratory function tests obtained at the time of bronchoscopy, we analyzed the items which were available at the time of diagnose. In particular, the neutrophil-to-lymphocyte ratio (NLR) in blood tests is widely used in several chronic inflammatory diseases [8–17]. However, there are few reports on the usefulness of the NLR in the BALF [18].

Baseline pulmonary function tests, including FVC and DLCO, were performed up to 3 months before bronchoscopy. HRCT scans were reviewed by a specialized pulmonologist, and the overall pattern was categorized as UIP, defined according to the 2018 American Thoracic Society/ European Respiratory Society/Japanese Respiratory Society/Asociación Latinoamericana de Tórax guidelines [6].

We also analyzed the 3-year mortality in PF-ILD and the parameters related to 3-year mortality. The median survival after diagnosis of IPF is reported to be 2 or 3 years [6]. In the case of IIPs, MCTD-ILDs, RA-ILDs, and SSc-ILDs, the median survival of PF-ILD patients is reported to range from 3.1 to 3.7 years [19]. Based on the above previous reports, we selected “3 years” as the survival duration in this study.

Patients eligible for data collection are those who underwent bronchoscopy between March and April 2018. The final follow-up timepoint is April 2021, after bronchoscopy in April 2018 and a 24-month follow-up as per Cottin’s definition. Alternatively, for cases diagnosed with PF-ILD at the 24-month follow-up period, death was evaluated 3 years after bronchoscopy.

### Statistical analysis

Data are presented as median (interquartile range). Data were compared between the two groups using Student’s *t*-test, the Mann–Whitney U test, and the χ^2^ test. Univariate and multivariate logistic regression analyses were performed to assess the effects of various factors on the diagnosis and survival of patients with PF-ILD. Items that differed significantly in the univariate analysis were selected and entered into the multivariate analysis. Receiver operating characteristic (ROC) curves were constructed to assess the areas under the curve (AUCs). The optimal cut-off values for predictors were determined by maximizing the Youden index. For all tests, two-sided *p*-values of <0.05 were considered statistically significant. Data were analyzed using JMP v16 (SAS Institute, Cary, NC, USA) and IBM SPSS^®^ Statistics version 27.0 (IBM SPSS Inc., Armonk, NY, USA).

## Results

### Baseline characteristics of the patients

The baseline characteristics of the study groups are shown in **Table 1**. Compared with the non-PF-ILD group, the PF-ILD group was older (median age 69.4 years) and was predominantly male. The PF-ILD group had a higher NLR, lower lymphocyte percentage (Lym%), lower CD8 levels, and higher CD4/CD8 ratio in the BALF. In addition, these patients showed a higher white blood cell count. No significant differences in blood fractions were observed between the groups.

**Table 1:** Baseline characteristics of the study groups.

	All	PF-ILD	Non-PF-ILD	*p*-value
	(n = 120)	(n = 77)	(n = 43)	
Follow-up duration, days (median, IQR)	1489.0 (1112.0–2394.0)	1431.5 (860.8–2154.5)	1593.0 (1224.0–2944.0)	0.076
Age, years (median, IQR)	69 (58.5–76)	71 (65–77)	65 (53–72)	0.003^a^
Male (%)	61 (50.8)	46 (59.7)	15 (34.9)	0.009^b^
BMI, kg/m^2^ (median, IQR)	22.5 (20.4–25.3)	23.4 (20.6–25.3)	21.1 (20.1–25.3)	0.203^a^
Smoking history (%)	71 (59.1)	49 (63.6)	22 (51.2)	0.155^b^
Smoking status (current) (%)	11 (9.2)	9 (11.7)	2 (4.7)	0.193^b^
Smoking, pack-year (median, IQR)	30 (15–50)	30 (15–50)	21 (10.5–47.5)	0.216^a^
**BALF (median, IQR)**				
Recovery rate of the BALF, %	40 (28.8–49.3)	39 (28.0–50.0)	42 (30.7–48.7)	0.414^a^
Cell Count (×10^5^/μL)	3(2–5)	3 (2–5)	4 (2–7)	0.228^a^
NLR	9.0 (1.8–41.1)	14.3 (2.7–50.0)	5.4 (1.1–20.9)	0.027^a^
MLR	205.4 (67.8–499.7)	285.3 (84.3–520.4)	116.5 (39.2–313)	0.058^a^
ELR	4.4 (0–16)	2.6 (0.0–21.7)	5.3 (0.0–12.3)	0.448^a^
BLR	0 (0.0–0.0)	0.0 (0.0–0.0)	0 (0.0–0.9)	0.729^a^
Neutrophils (%)	2.5 (0.5–9.0)	3.8 (0.6–10.2)	1.5 (0.5–6.0)	0.117^a^
Lymphocytes (%)	25.5 (12.5–49.5)	23.0 (11.4–41.9)	44 (19–64)	0.015^a^
Monocytes (%)	54.2 (25.9–77.5)	65.3 (26.6–78.0)	44.5 (25.5–71.5)	0.168^a^
Eosinophils (%)	1.5 (0.0–4.0)	1.0 (0.0–3.9)	2 (0.0–4.5)	0.074^a^
Basophils (%)	0 (0.0–0.0)	0.0 (0.0–0.0)	0.0 (0.0–0.5)	0.586^a^
CD4	54.9 (31.7–72.1)	56.7 (35.3–74.1)	42.9 (28.9–66.3)	0.056^a^
CD8	28.7 (17.8–55.3)	26.3 (16.0–36.7)	41.7 (24.0–66.9)	0.004^a^
CD4/CD8 ratio	1.9 (0.6–3.6)	2.1 (1.0–3.9)	1.0 (0.5–2.7)	0.021^a^
**Laboratory data (median, IQR)**				
White blood cell (/μL)	6900 (5500–9400)	7100 (6025–9600)	5900 (4400–8700)	0.026^a^
Neutrophils (%)	67.0 (57.5–74.5)	65.0 (60–72.8)	67.1 (56.4–76.1)	0.667^a^
Lymphocytes (%)	20.7 (15.3–29.9)	23.3 (17.0–29.9)	20.5 (14.6–29.2)	0.437^a^
Monocytes (%)	6.5 (5.1–8.3)	6.3 (5.4–8.6)	6.7 (5.4–8.6)	0.382^a^
Eosinophils (%)	3.4 (1.6–5.2)	3.4 (1.6–5.5)	0.5 (0.4–0.8)	0.488^a^
Basophils (%)	0.6 (0.4–0.9)	0.5 (0.4–0.8)	0.7 (0.4–0.9)	0.140^a^
NLR	3.1 (1.8–4.8)	3.2 (1.7–5.1)	2.8 (1.9–4.1)	0.812^a^
MLR	0.3 (0.2–0.5)	0.3 (0.2–0.5)	0.3 (0.2–0.5)	0.730^a^
ELR	0.1 (0.0–0.2)	0.1 (0.1–0.2)	0.2 (0.1–0.2)	0.850^a^
BLR	0.0 (0.02–0.04)	0.0 (0.0–0.0)	0.0 (0.0–0.0)	0.366^a^
Platelets (×10^4^/μL)	25.0 (20.2–30.1)	24.5 (19.7–29.2)	25.5 (22.5–31.6)	0.197^a^
CRP (mg/dl)	0.6 (0.2–3.9)	0.7 (0.2–4.8)	0.5 (0.1–2.8)	0.185^a^
LDH (U/L)	226 (195–286)	235 (194–291)	222 (194–274)	0.770^a^
KL-6 (U/ml)	837 (531–1580)	872 (589–1640)	724 (418–1433)	0.181^a^
**Management, n (%)**				
Steroid treatment or immunosuppressive treatment prior to BS	15 (12.5)	10 (13)	5 (11.6)	0.829^b^
Steroid treatment prior to BS	14 (11.7)	10 (13.0)	4 (9.3)	0.547^b^
Steroid treatment within 24 months of BS	74 (61.7)	46 (59.7)	28 (65.1)	0.561^b^
Immunosuppressive treatment within 24 months of BS	22 (18.3)	14 (18.2)	8 (18.6)	0.954^b^
Antifibrotic therapy within 24 months of BS	6 (5.0)	6 (7.8)	0 (0.0)	0.060^b^

**Abbreviations**: BMI: body mass index, NLR: neutrophil-to-lymphocyte ratio, MLR: monocyte-to-lymphocyte ratio, ELR: eosinophil-to-lymphocyte ratio, BLR: basophil-to-lymphocyte ratio, BS: bronchoscopy test, CRP: C-reactive protein, LDH: lactate dehydrogenase, IQR: interquartile range.

^a^ Mann–Whitney U test

^b^ Chi-square test.

Details of the ILD types that are present in the study population are shown in the S1 Table.

In the PF-ILD group, 10/77 (12.5%) patients were treated with steroids or immunosuppressive agents before bronchoscopy, which was similar to the proportion of patients in the non-PF-ILD group (5/43 [11.6%], *p* = 0.829). Immunosuppressive treatment included mizoribine, cyclophosphamide, cyclosporine, tacrolimus, azathioprine, and methotrexate. Of the patients, 46/77 (59.7%) started treatment with steroids and 14/77 (18.2%) started immunosuppressive treatment after bronchoscopy. There were no significant differences between the PF-ILD and non-PF-ILD groups with respect to the treatment used. In contrast, treatment with antifibrotic drugs was only initiated in the PF-ILD group (6/77, 7.8%).

### Diagnostic factors for PF-ILD

We performed univariate logistic regression analysis to assess the predictive markers of PF-ILD (Table 2). Male sex, older age, higher BALF NLR, higher BALF neutrophil percentage (Neu%), lower BALF Lym%, lower BALF eosinophil percentage (Eos%), and lower CD8 levels were diagnostic predictors of PF-ILD. The presence of an IPF/UIP pattern on HRCT had a higher odds ratio (OR) for PF-ILD (OR 19573899, *p* = 0.0007; Table 2). Steroid therapy or immunosuppressive treatment before bronchoscopy was not predictive of PF-ILD (OR 1.006, 95%CI 0.314–3.219, *p* = 0.092).

**Table 2:** Predictive Markers of PF-ILD according to univariate logistic regression analysis.

Parameter	Odds ratio	95%CI	*p*-value
Univariate			
**Sex (male)**	2.77	1.28–6.01	0.009
**Age**	1.04	1.01–1.08	0.003
**BALF NLR**	1.01	1.00–1.02	0.009
**BALF Neu%**	1.04	1.01–1.09	0.01
**BALF Lym%**	0.98	0.97–1.00	0.01
**BALF Eos%**	0.91	0.81–1.00	0.04
**CD8**	0.97	0.96–0.99	0.0014
**IPF/UIP pattern on CT scan**	19573899	-	0.092

**Abbreviations**: NLR: neutrophil-to-lymphocyte ratio; Neu%: neutrophil percentage; Lym%: lymphocyte percentage; Eos%: eosinophil percentage; BALF: bronchoalveolar lavage fluid.

**Fig 2:**
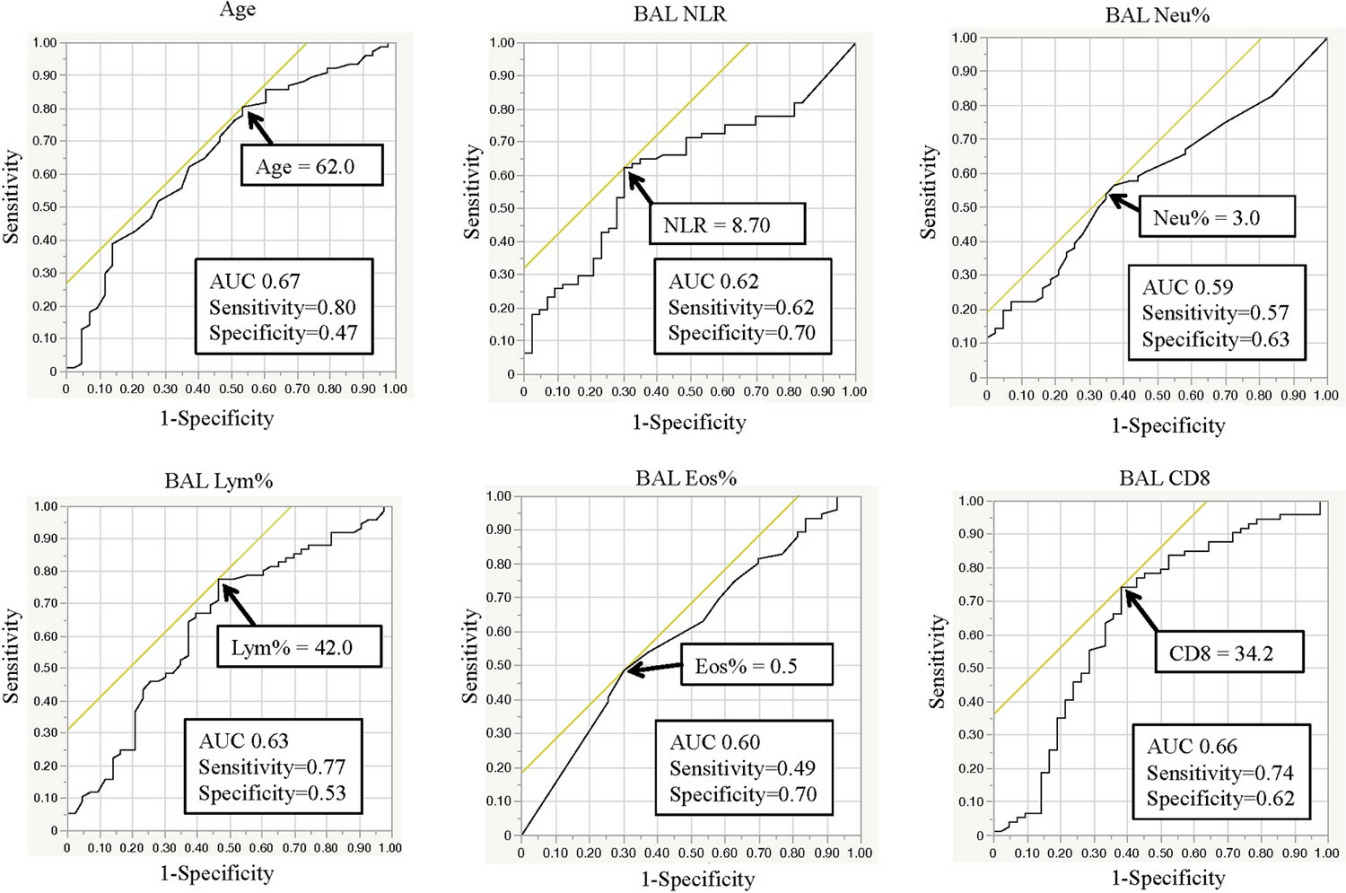
ROC curve analysis to establish cut-off values for PF-ILD diagnostic predictors. Abbreviations: NLR: neutrophil-to-lymphocyte ratio; AUC: area under the ROC curve; ROC: receiver operating characteristic.

For the predictive markers identified in the univariate analysis, we performed ROC curve analysis for continuous variables (Fig 2). Age, with a cut-off value of 62 years (0.80 sensitivity, 0.47 specificity, AUC 0.67); BALF NLR, with a cut-off value of 8.696 (0.62 sensitivity, 0.72 specificity, AUC 0.62); BALF Neu%, with a cut-off value of 3.0% (0.57 sensitivity, 0.63 specificity, AUC 0.59); BALF Lym%, with a cut-off value of 42.0% (0.77 sensitivity, 0.53 specificity, AUC 0.63); BALF Eos%, with a cut-off value of 0.5% (0.49 sensitivity, 0.70 specificity, AUC 0.60); and BALF CD8, with a value of 34.2 (0.74 sensitivity, 0.62 specificity, AUC 0.66), were identified as diagnostic predictors for PF-ILD.

We performed multivariate logistic regression analysis of three models using these predictive markers (**Table 3**). Cellular analysis of the BALF (NLR ≥ 8.696, Neu% ≥ 3%, Lym% ≤ 42%) and CD8 ≥ 34.2 at the time of bronchoscopy were found to be independent predictive markers of PF-ILD. Eos% was no longer significant in the multivariate analysis. The results did not differ even when the presence of the UIP pattern on HRCT was included.

**Table 3:** Predictive markers of PF-ILD based on multivariate logistic regression analysis.

**Multivariate analysis model 1**			
**Parameter**	**Odds ratio**	**95%CI**	***p*-value**
Sex (male)	2.017	0.806–5.051	0.134
Age ≥ 62 years	3.671	1.321–10.206	0.013
BALF NLR ≥ 8.696	6.252	2.327–16.795	0.0002
CD8 ≤ 34.2	4.436	1.715–11.474	0.002
**Multivariate analysis model 2**			
**Parameter**	**Odds ratio**	**95%CI**	***p*-value**
Sex (male)	2.240	0.927–5.414	0.073
Age ≥ 62 years	2.922	1.138–7.502	0.026
BALF Neutrophil% ≥ 3.0	3.206	1.295–7.934	0.012
CD8 ≤ 34.2	4.286	1.738–10.573	0.002
**Multivariate analysis model 3**			
**Parameter**	**Odds ratio**	**95%CI**	***p*-value**
Sex (male)	2.017	0.825–4.926	0.124
Age ≥ 62 years	3.197	1.211–8.44	0.019
BALF Lymphocyte% ≤ 42.0	3.942	1.561–9.955	0.004
CD8 ≤ 34.2	3.358	1.365–8.258	0.008

We selected several parameters: model 1, model 2, and model 3.

**Abbreviations**: NLR: neutrophil-to-lymphocyte ratio; BALF: bronchoalveolar lavage fluid; CI, confidence interval.

### Prognostic factors of 3-year mortality among patients with PF-ILD

Next, we explored the predictors of 3-year mortality in the PF-ILD group. Univariate logistic regression analysis showed that high lactate dehydrogenase (LDH), high Krebs von den Lungen-6 (KL-6), low baseline FVC on respiratory function tests, and high BALF Neu% were predictors of death within 3 years. The presence of an IPF/UIP pattern on the HRCT scan was also a predictor of 3-year mortality (**Table 4**).

**Table 4:** Predictive markers of death in the PF-ILD group within 3 Years, based on univariate logistic regression analysis.

Parameter	Odds ratio	95%CI	*p*-value
Univariate			
**LDH**	1.008	1.000–1.016	0.047
**KL-6**	1.001	1.000–1.002	0.024
**FVC baseline**	3.286	1.161–9.297	0.025
**BALF Neu%**	1.03	1.003–1.059	0.032
**IPF/UIP pattern on CT scan**	11.00	2.272–53.266	0.003

**Abbreviations**: LDH: lactate dehydrogenase; KL-6: Krebs von der Lungen-6; FVC: forced vital capacity; BALF: bronchoalveolar lavage fluid; Neu%: neutrophil percentage.

**Fig 3:**
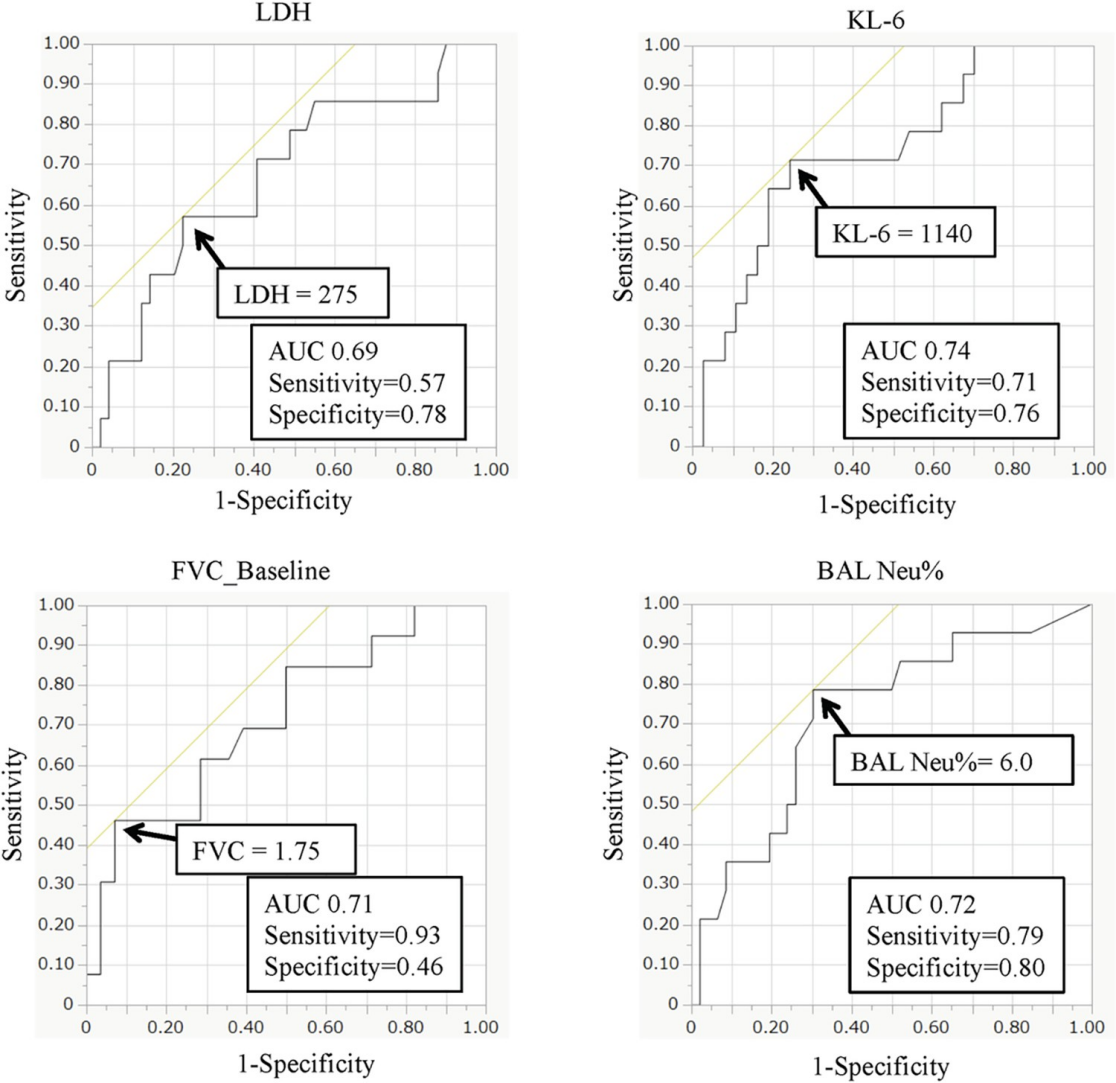
ROC curve analysis of the predictor cut-off values for 3-year mortality. Abbreviations: AUC: area under the ROC curve; ROC: receiver operating characteristic.

As mentioned before, we performed ROC curve analysis for the continuous variables: LDH, KL-6, baseline FVC values, and BALF Neu% (Fig 3). Predictors of 3-year mortality in the PF-ILD group were LDH ≥ 274 U/L, KL-6 ≥ 1110 U/ml, baseline FVC ≤ 1.79 L, and BALF Neu ≥ 5.25%. For KL-6, FVC, and BALF Neu%, the AUC exceeded 0.7, which could be interpreted as indicating relatively high accuracy.

Multivariate logistic regression analysis using these predictors showed that BALF Neu% ≥ 5.25% and baseline FVC < 1.79 L at bronchoscopy were independent predictors of 3-year mortality (BALF Neu%: OR 11.467, 95%CI 1.151–128.023, *p* = 0.038; FVC baseline: OR 37.052, 95%CI 2.193–625.929, *p* = 0.012) in the PF-ILD group (**Table 5**). The results did not differ even when the presence of the UIP pattern on HRCT was included in this analysis.

**Table 5:** Predictive markers of death within 3 years in the PF-ILD group, based on multivariate logistic regression analysis.

Multivariate analysis			
Parameter	Odds ratio	95%CI	*p*-value
**BALF Neu% ≥ 6 (%)**	**11.467**	**1.091–120.532**	**0.042**
**LDH ≥ 275 (U/L)**	3.067	0.274–34.286	0.363
**FVC baseline ≤ 1.75 L**	3.896	0.437–34.735	0.223
**KL-6 ≥ 1140 (U/ml)**	37.052	2.193–625.929	0.012

**Abbreviations**: LDH: lactate dehydrogenase; KL-6: Krebs von der Lungen-6; FVC: forced vital capacity; BALF: bronchoalveolar lavage fluid; Neu%: neutrophil percentage; CI: confidence interval.

## Discussion

The diagnosis of PF-ILD has been based on imaging findings, clinical symptoms, and respiratory function test results, and a time course investigation is required to distinguish it from ILDs other than IPFs [7]. Respiratory function tests are now less commonly performed than before due to the coronavirus disease 2019 pandemic. In this study, we sought to identify biomarkers for the diagnosis and prognosis of PF-ILD. Our study revealed predictive markers of PF-ILD using only blood tests and the BALF obtained at the bronchoscopic evaluation. As diagnostic predictors, we identified age, NLR, and CD8 levels in the BALF. As prognostic markers, we identified serum LDH, KL-6, FVC, and BALF Neu%.

NLR based on blood tests is an inexpensive and widely available marker of chronic inflammation. The usefulness of the NLR in the blood has been reported in patients with stroke [9], cancer [10], cardiovascular diseases [11], hypertension [11], sepsis [12], diabetes mellitus [13], hepatic cirrhosis [14], rheumatoid arthritis [15], chronic obstructive pulmonary disorder [17], and asthma [8]. However, there are few reports on its usefulness in the BALF [18]. In our study, the NLR in the BALF, but not that in the blood, was associated with PF-ILD diagnosis. The difference in the usefulness of the NLR between the BALF and blood suggests that the BALF reflects local inflammation, whereas the blood reflects systemic inflammation.

CD8^+^ T cells in the BALF were also associated with the diagnosis of PF-ILD in this study. Daniil et al. reported that increased CD8^+^ T lymphocytes in the lung biopsies of patients with IPF correlated with decreased lung function [20]. In a bleomycin-induced pulmonary fibrosis model, CD8^+^ T cells were shown to differentiate into profibrotic IL-13-producing cells [21]. These data suggested that CD8^+^ T cells are associated with lung fibrosis.

According to the guidelines related to IPF, lymphocyte counts in the BALF of patients with IPF are lower than those in patients with non-IPF interstitial pneumonia [22]. Takei et al. reported that the differential lymphocyte count in the BALF was a prognostic factor for acute exacerbation in patients with chronic fibrosing IIPs [23].

Kinder et al. reported that the Neu% in the BALF correlated with mortality in patients with IPF [24]. In acute exacerbation of IPF, neutrophils in the BALF are a poor prognostic factor [25]. However, neutrophil depletion in rats and mice did not protect against bleomycin-induced pulmonary fibrosis [26]. Therefore, the role of neutrophils in lung fibrosis remains unclear. Nevertheless, our study supports the significance of neutrophils in patients with PF-ILD.

Currently, for the diagnosis of PF-ILD, appropriate treatment should have been administered within 24 months of diagnosis. In our study population, approximately 60% of the PF-ILD group was treated with steroids and approximately 18% were treated with immunosuppressive drugs within 24 months of diagnosis (Table 1), which was similar to the numbers reported in a previous UK test and validation cohort [27].

Although bronchoscopy is not necessary for patients with a UIP pattern on CT scan, patients with IPF/UIP had a higher neutrophil percentage in their BALF [6]. Furthermore, patients with hypersensitivity pneumonitis (HP) or sarcoidosis had a higher lymphocyte percentage in their BALF [6]. S1 Table shows the analysis of BAL samples and detailed diseases. Sarcoidosis was included in the IIPs/non-IPF group. The lymphocyte count in the BALF was significantly higher in the HP group than in the IPF and IIPs groups (mean [IQR], HP group: 56.5 [32.0–77.5], IPF group: 16.35 [8.0–42.5], IIPs group: 25.5 [9.5–46.75]; p value [one-way ANOVA]: 0.014). The CD4 count in the BALF was higher in the IPF group than in the CTD-ILD group (mean [IQR], IPF group: 72.6 [63.7–75.7], CTD-ILD group: 33.3 [22.8–55.3]; p value [one-way ANOVA]: 0.010). The CD8 count in the BALF was higher in the CTD-ILD group than in the IPF and IIPs groups (mean [IQR], CTD-ILD group: 55.2 [31.5–65.2], IPF group: 21 [15.9–24.8], IIPs group: 27 [15.1–39]; p value [one-way ANOVA]: 0.005). Both CTD-ILD and HP are pathologies that may or may not progress to PF-ILD.

The purpose of our study was to search for factors that could be used to diagnose PF-ILD at the time of bronchoscopy, irrespective of the final name of the disease. Nintedanib is approved for PF-ILD. Flaherty et al. showed that the annual rate of decline in the FVC was significantly lower among patients with PF-ILD who received nintedanib than among those who received placebo [28]. In our study, six patients received antifibrotic drugs after bronchoscopy, and all were in the PF-ILD group. There may be a difference in the 3-year mortality between patients who were treated with antifibrotic drugs, including nintedanib, and those who were not. Diagnosing PF-ILD at an early stage and ensuring initiation of antifibrotic drugs at an appropriate time is important, and we hope that this study will help in this regard.

There are some potential limitations to the present study. First, the number of enrolled patients was small, and similar investigations should be performed using larger sample sizes. Second, this was a retrospective study conducted at a single center, indicating a potential risk of selection and recall biases. Third, the HRCT scans were reviewed by the person involved in the data analysis of this study; however, we have reviewed the HRCT scans prior to data analysis to try to avoid bias as much as possible.

In conclusion, this study revealed diagnostic and prognostic factors for patients with PF-ILD based on BALF, blood, and respiratory function tests. In particular, cellular analysis of BALF may be useful in the diagnosis and prognosis prediction of PF-ILD.

## Supporting information

S1 Table Details of the ILD types. Among the PF-ILD group, 10 patients had IPF, 37 had IIPs/non IPF, 13 had CTD-ILD, 12 had HP, 1 had drug-induced lung injury, and 4 had other IIPs including pleuroparenchymal fibroelastosis (PPFE), acute interstitial pneumonia (AIP), and unclassifiable IPF.
(XLSX)
